# Supplementary material for: Endocannabinoid signaling regulates the reinforcing and psychostimulant effects of ketamine in mice
Source: Nat Commun. 2020 Nov 24;11:5962. doi: 10.1038/s41467-020-19780-z (PMC7686380; doi:10.1038/s41467-020-19780-z)
Supplement: Supplementary file 1 — Supplementary Information [file 41467_2020_19780_MOESM1_ESM.pdf]

Cover Page – Supplementary Information

Endocannabinoid signalling regulates the reinforcing and psychostimulant effects of ketamine in mice

Wei Xu, et al.

Supplementary figures

Supplementary Figure 1

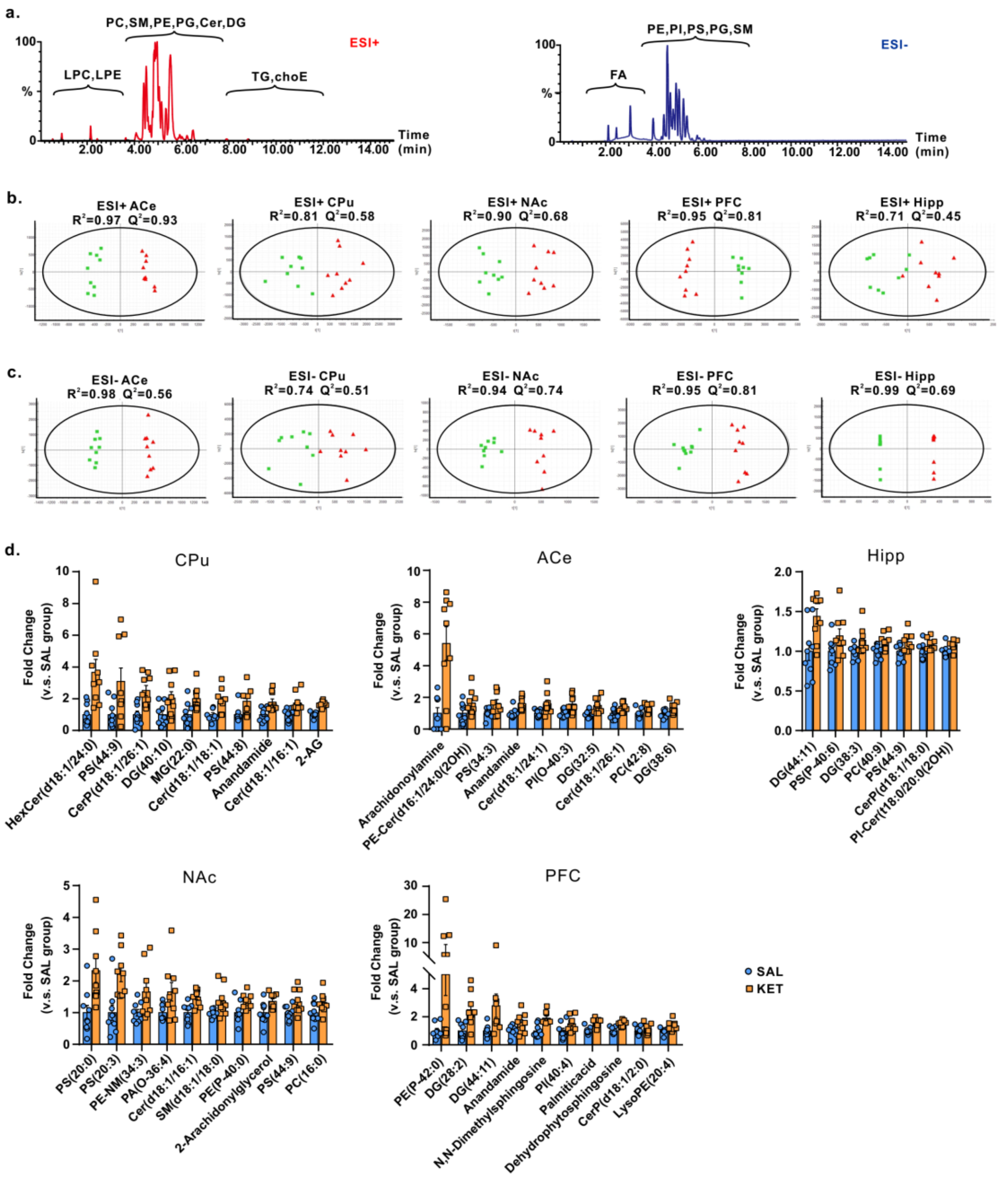

Supplementary figure 1. Ketamine modifies brain lipidome. **(a)** Typical UPLC/MS base peak ion chromatogram of the lipids extracted from mice brain using electrospray ionization in the positive (ESI+) and negative (ESI-) ion mode respectively. **(b)** OPLS-DA analysis under ESI positive detection modes. **(c)** OPLS-DA analysis under ESI negative detection modes. The model parameters R^2 explained variance and Q^2 indicated the excellent quality of model. ACe, central amygdaloid nucleus. CPu, caudate nucleus and putamen. NAc, nucleus accumbens. PFC, prefrontal cortex. Hipp, hippocampus. **(d)** Top 10 lipids with the most significant elevation in the brain region. Data are shown as mean ± SEM. Source data provided as a Source Data file.

Supplementary Figure 2

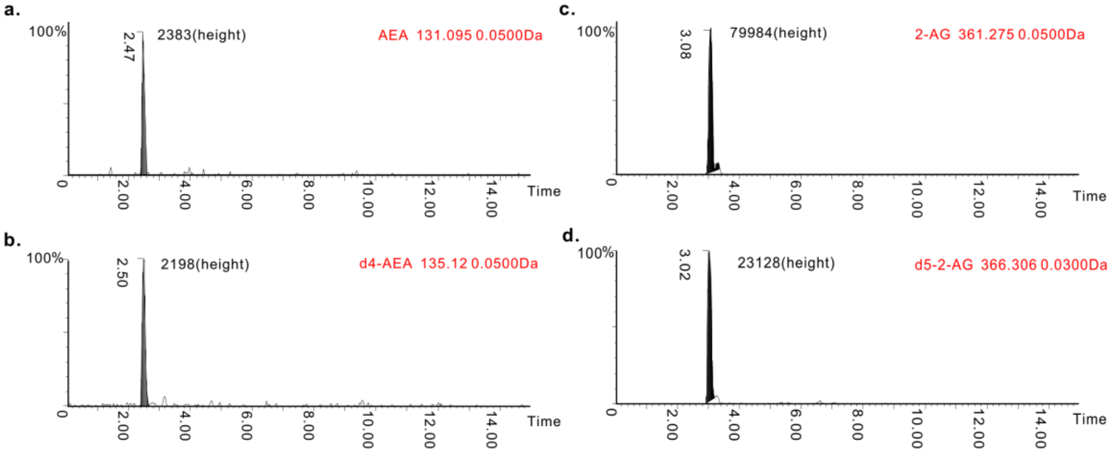

Supplementary figure 2. Representative LC–MS chromatograms of analytes in the CPu. **(a)** AEA, retention time (RT) 2.47 min, daughter mass 131.0946 Da. **(b)** d4-AEA, RT 2.50 min, daughter mass 135.1197 Da. **(c)** 2-AG, RT 3.08 min, daughter mass 361.2748 Da. (d) d5-2-AG, RT 3.02 min, daughter mass 366.3057 Da. RT difference should be within 0.1 min and the mass difference should be less than 0.1 Da between detection and prediction.

Supplementary Figure 3

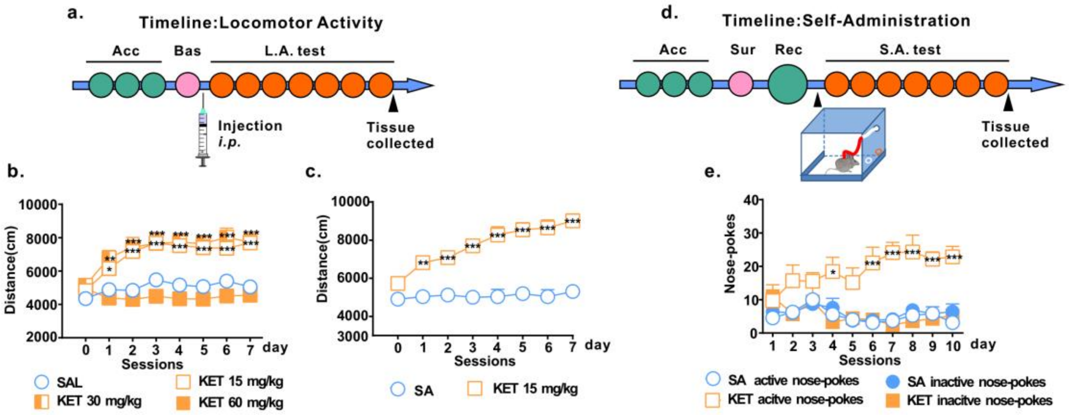

Supplementary figure 3. Ketamine's effect on mice behavior. **(a)** Experimental time course for locomotor activity test. **(b)** Hyperlocomotion effect of variant regimen of ketamine (15, 30, 60 mg/kg) (n=10 mice/group, two-way repeated measured ANOVA, followed by Dunnett's multiple comparisons test, Treatment F(3,36)=33.53, *P*<0.0001; Time F(7,252)=11.62, *P*<0.0001; Interaction F(21,252)=3.333, *P*<0.0001). **(c)** 15 mg/kg ketamine significantly increased the locomotor activity in mice (n=12 mice/group, two-way repeated measured ANOVA, followed by Bonferroni's multiple comparisons test, Drug F(1, 22)=74.85, *P*<0.0001; Time F(7,154)=9.206, *P*<0.0001; Interaction F(7, 154)=6.744, *P*<0.0001). **(d)** Experimental time course for ketamine self-administration. **(e)** Ketamine significantly increased active nosepokes without affecting inactive nosepokes (n=7 mice/group, Active nosepokes: two-way ANOVA, followed by Bonferroni's multiple comparisons test, Drug F(1,12)=109.4, *P*<0.0001; Time F(9,108)=1.325, *P*=0.2324; Interaction F(9,108)=2.077, *P*=0.0378). Data are shown as mean ± SEM. Compared to SAL group, **P*<0.05, ***P*<0.01, ****P*<0.001. SAL, saline; KET, ketamine. Source data and statistics provided as a Source Data file.

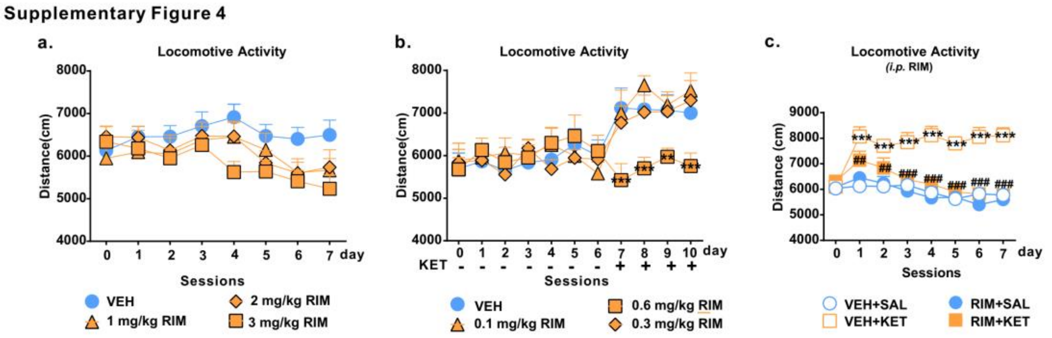

Supplementary figure 4. Rimonabant attenuates ketamine-induced hyperlocomotor activity in mice. **(a)** Effect of 1~3 mg/kg rimonabant (*i.p.*) on locomotor activity in mice. n=11 mice/group. Two-way repeated measured ANOVA, followed by Dunnett's multiple comparisons test, dose F(3,30)=1.044, *P*=0.3876; Time F(7,70)=7.989, *P*<0.0001; Interaction F(21,210)=1.733, *P*=0.0279. **(b)** 0.1~0.6 mg/kg rimonabant (*i.p.*) did not affect the baseline locomotor activity in mice; 0.6 mg/kg rimonabant inhibited ketamine-induced hyperlocomotion. n=10 mice/group. Two-way repeated measured ANOVA, followed by Dunnett's multiple comparisons test, dose F(3,27)=0.6105, *P*=0.6140; Time F(10,90)=17.872, *P*<0.0001; Interaction F(30,270)=3.657, *P*<0.0001. **(c)** 0.6 mg/kg rimonabant (*i.p.*) reduced ketamine-induced hyperlocomotion in mice (n=14 mice/group. Two-way repeated measured ANOVA, followed by Dunnett's multiple comparisons test, Treatment F(3,39)=25.99, *P*<0.0001; Time F(7,91)=7.835, *P*<0.0001; Interaction F(21,273)=4.403, *P*<0.0001). Compared to VEH+SAL group, ****P*<0.001; Compared to VEH+KET, ##*P*<0.01, ###*P*<0.001. Data are shown as mean ± SEM. SAL, saline; KET, ketamine; VEH, vehicle; RIM, rimonabant. Source data and statistics provided as a Source Data file.

Supplementary Figure 5

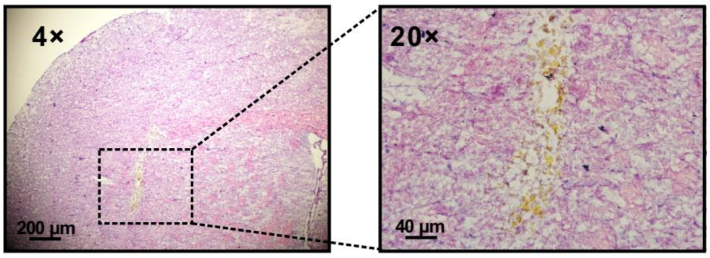

Supplementary figure 5. Representative images of intra-cannulation in the DLS. The outer catheter was accurately implanted in the DLS without inducing inflammatory reaction.

Supplementary Figure 6

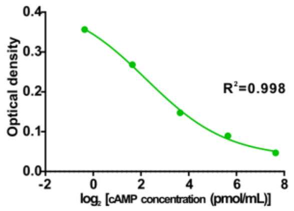

Supplementary figure 6. The standard curve for cAMP concentration detection. Four parameter fitting method was used for standard curve fitting. R^2=0.998. Source data provided as a Source Data file.

Supplementary Figure 7

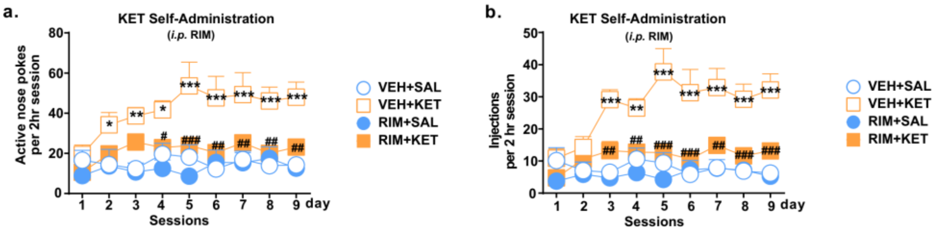

Supplementary figure 7. Rimonabant reduces ketamine self-administration. (a) 0.6 mg/kg rimonabant (*i.p.*) attenuated ketamine-seeking behavior (n=8 mice/group. Two-way repeated measured ANOVA, followed by Dunnett's multiple comparisons test, Treatment $F(3,28)=11.6$, $P<0.001$; Time $F(8,224)=3.862$, $P=0.0003$; Interaction $F(24,224)=1.794$, $P=0.0156$). **(b)** 0.6 mg/kg rimonabant (*i.p.*) decreased ketamine-taking behavior (n=8 mice/group. Two-way ANOVA, followed by Dunnett's multiple comparisons test, Treatment $F(3,28)=17.08$, $P<0.0001$; Time $F(8,224)=5.554$, $P<0.0001$; Interaction $F(24,224)=3.37$, $P<0.0001$). Data are shown as mean ± SEM. Compared to VEH+SAL group, *$P<0.05$, **$P<0.01$, ***$P<0.001$; Compared to VEH+KET, #$P<0.05$, ##$P<0.01$, ###$P<0.001$. SAL, saline; KET, ketamine; VEH, vehicle; RIM, rimonabant. Source data and statistics provided as a Source Data file.

Supplementary Figure 8

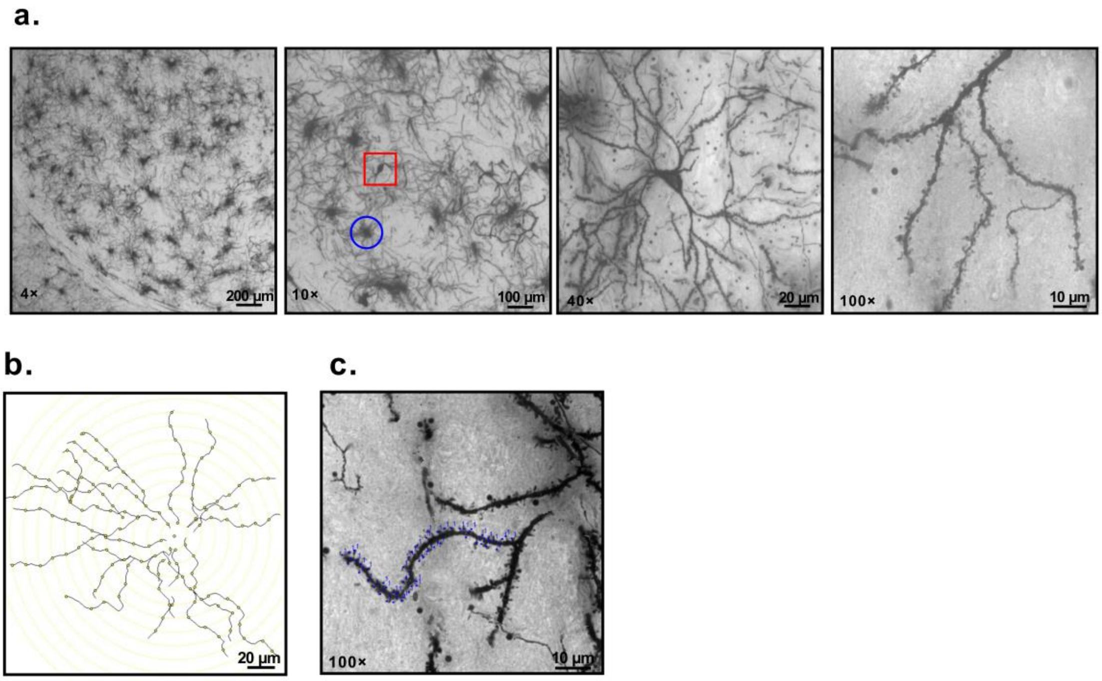

Supplementary figure 8. Golgi-Cox staining and analysis. **(a)** Representative images of Golgi-Cox staining of DLS under 4×, 10×, 40× and 100× lens, respectively. Red rectangle shows a spiny projection neuron, and blue circle shows an astrocyte. **(b)** Representative image for intersection analysis with Sholl software. Stepsize=10 μm. **(c)** Representative image for dendrite counting with Image J software.

Supplementary Figure 9

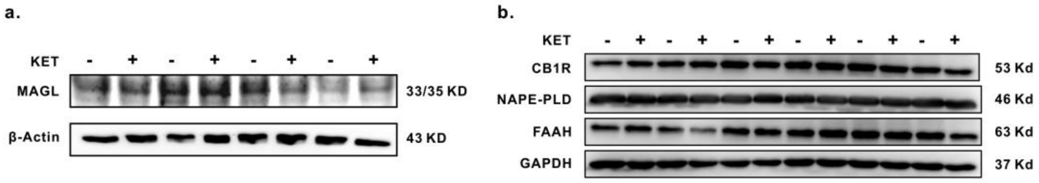

Supplementary figure 9. The effects of ketamine on ECS in the PFC and CPu. **(a)** Ketamine didn't affect MAGL expression in the PFC. n=4 mice/group. **(b)** Ketamine did not alter the expression of CB1R and AEA metabolic enzymes in the CPu. n=6 mice/group. KET, ketamine; NAPE-PLD, N-acyl phosphatidylethanolamine phospholipase D; FAAH, fatty acid amide hydrolase.

Supplementary Figure 10

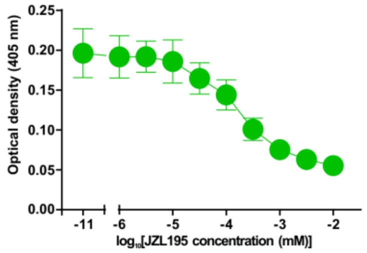

Supplementary figure10. JZL195 (positive control) significantly inhibits MAGL activity. n=3 samples/concentration. Data are shown as mean ± SEM. Source data and statistics provided as a Source Data file.

Supplementary Figure 11

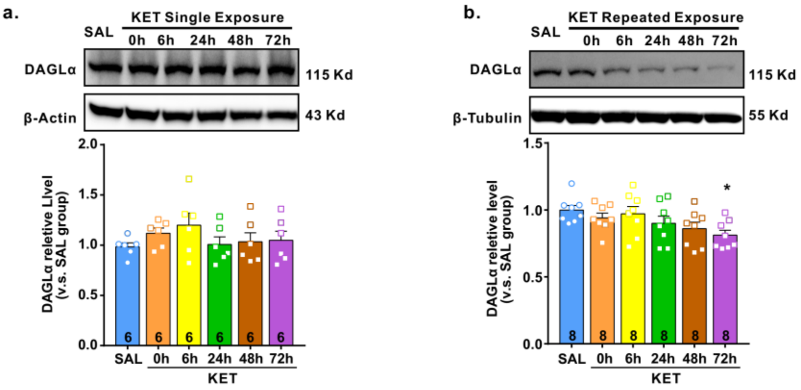

Supplementary figure 11. The effect of ketamine on DAGLα expression. (a) DAGLα expression was not altered by a single ketamine injection (15 mg/kg) (One-way ANOVA, followed by Dunnett's multiple comparisons test, $F(5,30)=0.9481$, $P=0.4647$. **(b)** DAGLα expression was only decreased at 72 hours after repeated ketamine exposure (15 mg/kg) (One-way ANOVA, followed by Dunnett's multiple comparisons test, MAGL $F(5,42)=2.467$, $P=0.0478$; 72 h vs. SAL, $P=0.0217$). Compared to SAL group, $^*P<0.05$. Data are shown as mean ± SEM. SAL, saline; KET, ketamine. Source data and statistics provided as a Source Data file.

Supplementary Figure 12

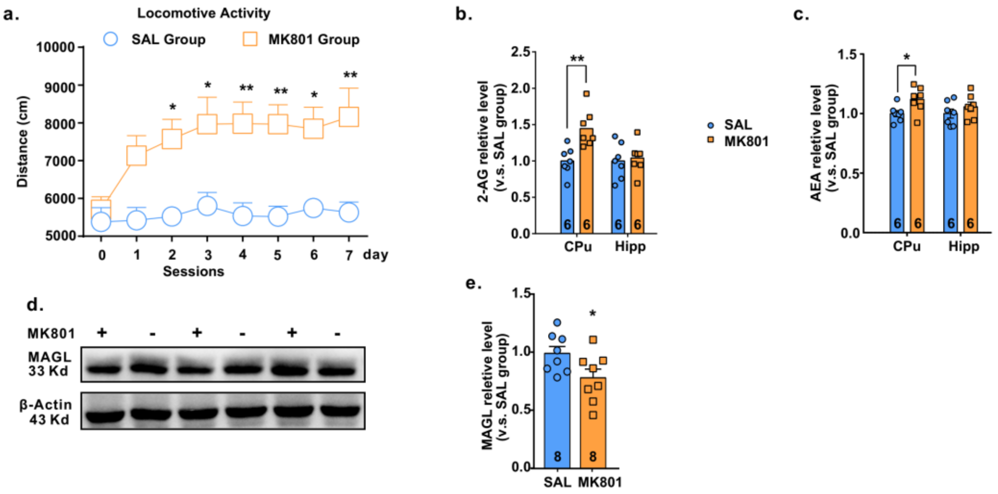

Supplementary figure 12. MK801 modulates eCB signalling in the CPu. **(a)** MK801 (0.3 mg/kg, *i.p.*) resulted in hyperlocomotion in mice (n=8 mice/group. two-way repeated measured ANOVA, followed by Bonferroni's multiple comparisons test, Drug F(1, 12)=14.02， *P*=0.0022；Time F(7, 98)=4.716，*P*=0.0001；Interaction F(7, 98)=2.908, *P*=0.0083). **(b)** MK801 increased 2-AG level in the CPu (Unpaired two-tailed *t* test, in CPu t(12)=3.655, *P*=0.0033; in Hipp, t(12)=0.3333, *P*=0.7447). **(c)** MK801 increased AEA level in the CPu (Unpaired two-tailed *t* test, in CPu t(12)=2.476, *P*=0.0291; in Hipp, t(12)=1.076, *P*=0.3029). **(d, e)** MK801 (0.3 mg/kg) decreased MAGL expression in the CPu (Unpaired two-tailed *t* test, t(14)=2.195, *P*=0.0456). Data are shown as mean ± SEM. SAL, saline; KET, ketamine. Source data and statistics provided as a Source Data file.

Supplementary Figure 13

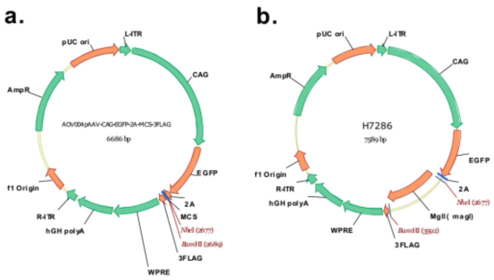

Supplementary figure 13. The construction diagram of *Mgll* and eGFP negative plasmids (mice). **(a)** The plasmid expresses eGFP. **(b)** The plasmid expresses *Mgll* (mice).

Supplementary Figure 14

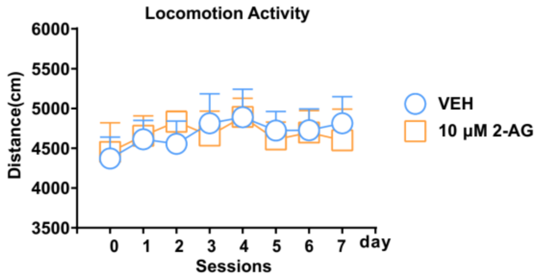

Supplementary figure 14. Intra-DLS 2-AG supplement has no effect on the baseline locomotion in mice. 2-AG: 10 μM, 1 μL per side. n=9 mice/group. Two-way repeated measured ANOVA, Treatment F(1,16)=0.0024, *P*=0.0962, Time F(7,112)=0.6053, *P*=0.7505, Interaction F(7,112)=0.2051, *P*=0.9837. Data are shown as mean ± SEM. Source data and statistics provided as a Source Data file.

Supplementary Figure 15

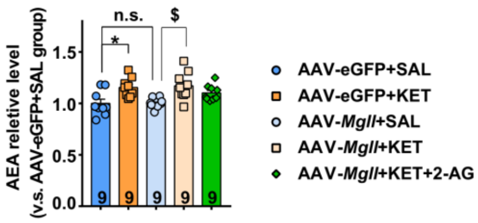

Supplementary figure 15. Intra-DLS infusion of AAV-*Mgll* has no effect on DLS AEA level. n=9 mice/group. One-way ANOVA, followed by Tukey's multiple comparisons test, F(4,40)=5.827, *P*=0.0009; AAV-eGFP+KET v.s. AAV-eGFP+SAL, *P*<0.05; AAV-*Mgll*+SAL v.s. AAV-eGFP+SAL, *P*>0.05; AAV-*Mgll*+KET v.s. AAV-*Mgll*+SAL, *P*<0.05. Compared to AAV-eGFP+SAL group, **P*<0.05; Compared to AAV-*Mgll*+SAL group, $*P*<0.05. Data are shown as mean ± SEM. SAL, saline; KET, ketamine. Source data and statistics provided as a Source Data file.

Supplementary Figure 16

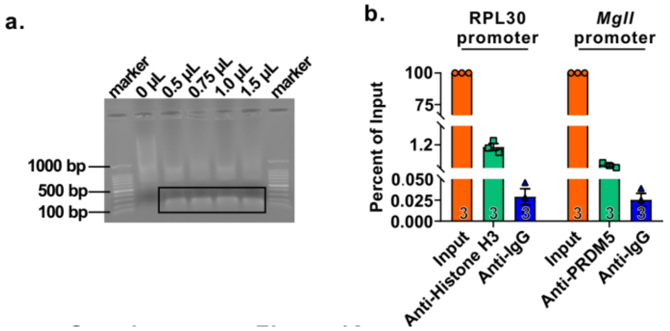

Supplementary figure 16. Enzyme digestion and enrichment efficiency tests of ChIP. (a) Analysis of chromatin digestion with micrococcal nuclease. The majority of chromatin from brain was digested to 1 to 5 nucleosomes in length (150 to 900 bp) by 0.5-1μL micrococcal nuclease. **(b)** Enrichment efficiency of histone H3 in the promoter of RPL30 gene (positive control) and enrichment efficiency of PRDM5 in the promoter of *Mgll* gene. The high enrichment efficiency of histone H3 and PRDM5 with low IgG enrichment represented good signal-to-noise ratio. n=3 samples/group. Data are shown as mean ± SEM.

Supplementary Figure 17

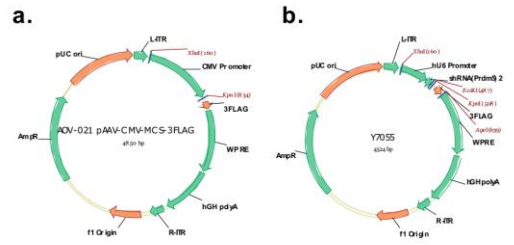

Supplementary figure 17. The construction diagram of *Prdm5*-shRNA (mice) and scramble-shRNA plasmids for AAV. (a) The plasmid expresses scramble-shRNA. **(b)** The plasmid expresses *Prdm5*-shRNA (mice).

Supplementary Figure 18

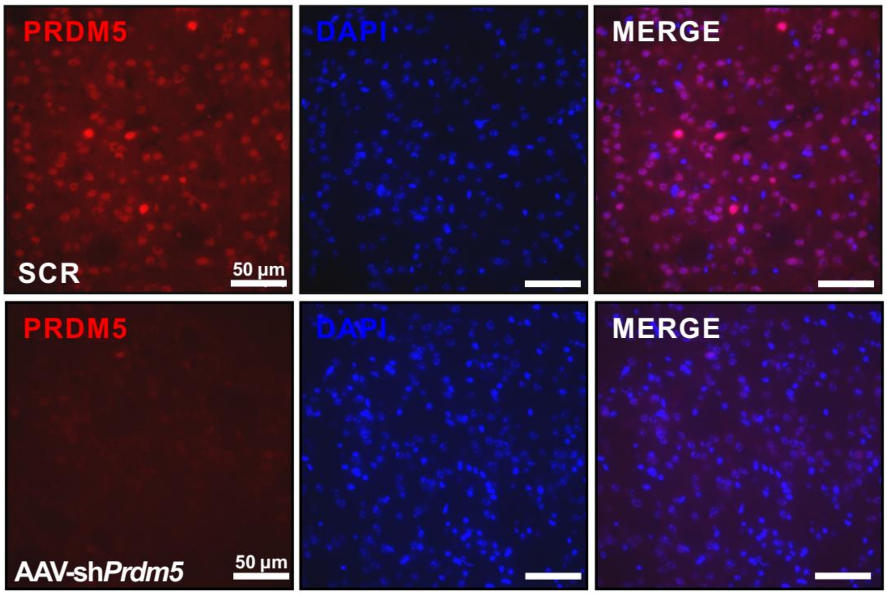

Supplementary figure 18. Representative images of silence efficiency detection for AAV-*shPrdm5.* AAV-*shPrdm5* effectively silences PRDM5 expression (red).

Supplementary Figure 19

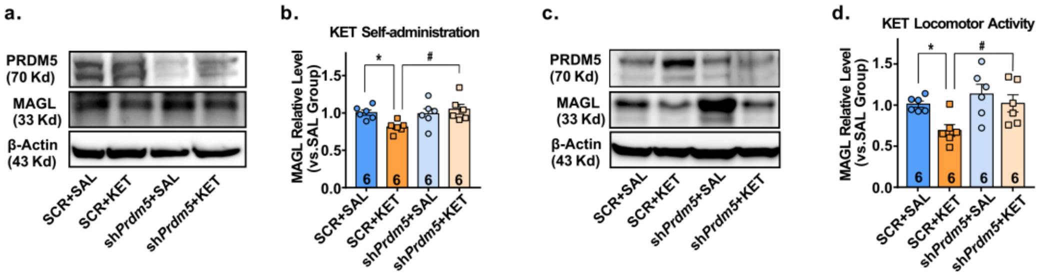

Supplementary figure 19. AAV-*shPrdm5* increases MAGL expression in the DLS in two ketamine addiction paradigms. **(a, b)** AAV-*shPrdm5* promoted MAGL expression in DLS under ketamine self-administration paradigm (One-way ANOVA, followed by Dunnett's multiple comparisons test, F(3,20)=3.867, *P*=0.0248; SCR+KET group v.s. SCR+SAL group, *P*=0.0479; AAV-sh*Prdm5*+KET group v.s. SCR+KET group, *P*=0.0131). **(c, d)** AAV-*shPrdm5* enhanced the MAGL expression in the DLS under ketamine hyperlocomotion paradigm (One-way ANOVA, followed by Dunnett's multiple comparisons test, F(3,20)=4.583, *P*=0.0134; SCR+KET group v.s. SCR+SAL group, *P*=0.0492; AAV-sh*Prdm5*+KET group v.s. SCR+KET group, *P*=0.0426). Data are shown as mean ± SEM. Compared to SCR+SAL group, **P*<0.05; Compared to VEH+KET, #*P*<0.05. SAL, saline; KET, ketamine; SCR, scrambled RNA. Source data and statistics provided as a Source Data file.

Supplementary Figure 20

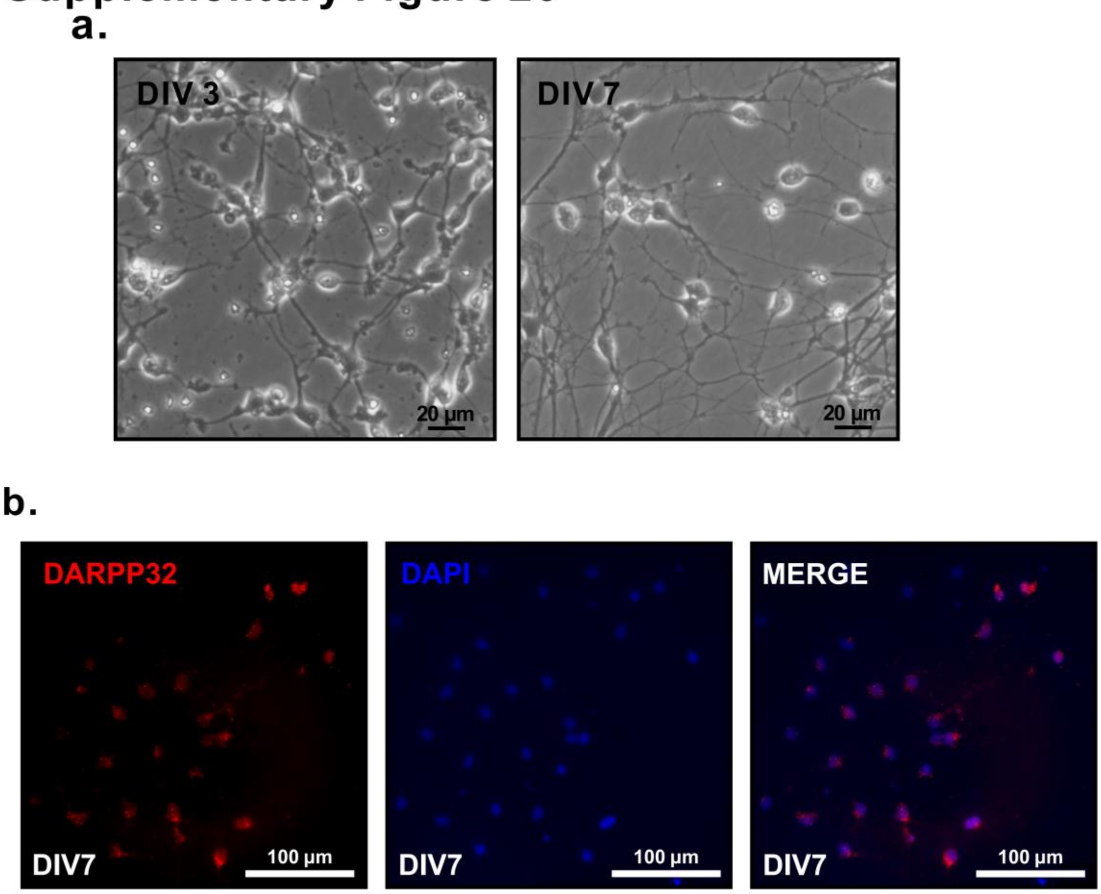

Supplementary figure 20. Representative images of the primary cultured SPNs. (a) Representative images of the primary cultured SPNs at DIV3 and DIV7, respectively. SPNs began making contacts to form a neural net at DIV3 and became closely connected at DIV7. **(b)** SPNs were the predominant type of cells in the culture, as evidenced by DARPP32 immunostaining.

Supplementary Figure 21

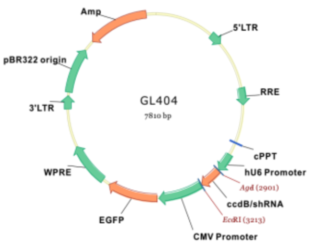

Supplementary figure 21. The construction diagram of shRNA (rat) plasmids for lentivirus. For *Prdm5*-shRNA (rat) plasmid, *Prdm5*-shRNA (rat) was promoted by hU6 promoter and eGFP was promoted by CMV promoter.

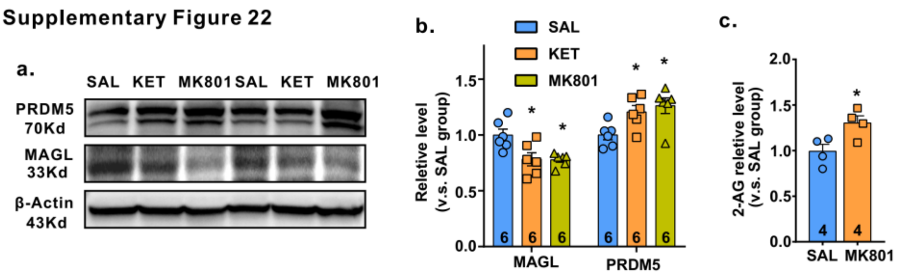

Supplementary figure 22. **(a, b)** Both MK801 and ketamine decreased MAGL but increased PRDM5 expression in the primary cultured SPN (One-way ANOVA, followed by Dunnett's multiple comparisons test, MAGL F(2,15)=6.907, *P*=0.0075; PRDM5 F(2,15)=5.731, *P*=0.0142). **(c)** MK801 increased 2-AG level in the SPN (Unpaired two-tailed *t* test, t(6)=2.809, *P*=0.0308). Data are shown as mean ± SEM. Compared to SAL group, **P*<0.05, ***P*<0.05. SAL, saline; KET, ketamine. Source data and statistics provided as a Source Data file.

Supplementary Figure 23

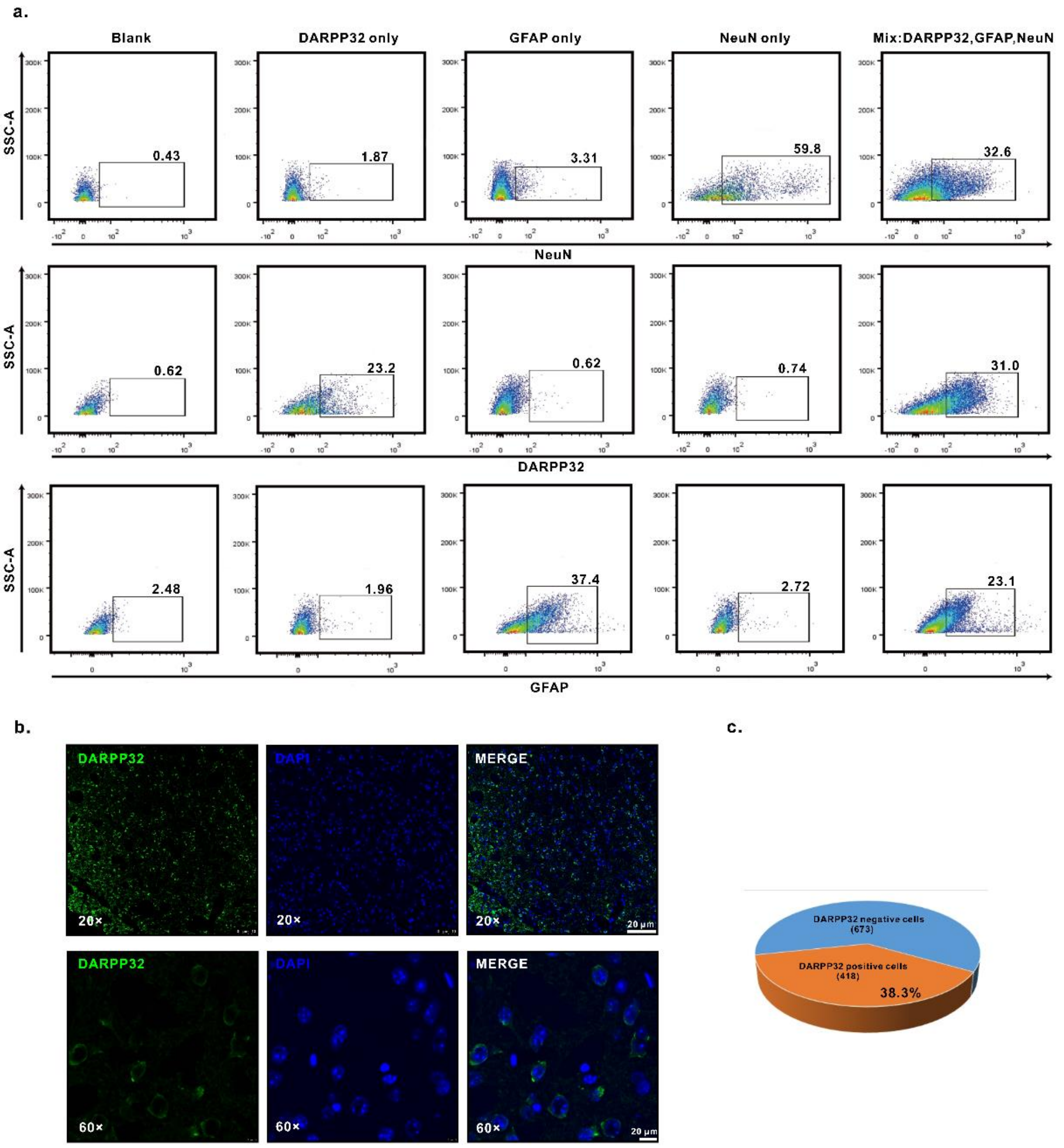

Supplementary figure 23. Quantitative analysis of neuron subpopulations in the CPu. **(a)** The ratio of DARPP32-positive cells was ~31% in the CPu (detected by fluorescence-activated cell sorting). SSC-A: side scatter area, indicating the cell density and complexity. The quadrants show the corresponding positive subpopulations. **(b)** Representative images of DARPP32 positive cells. **(c)** The ratio of DARPP32-positive cells was ~38% in the CPu (detected by immunofluorescence).

Supplementary Figure 24

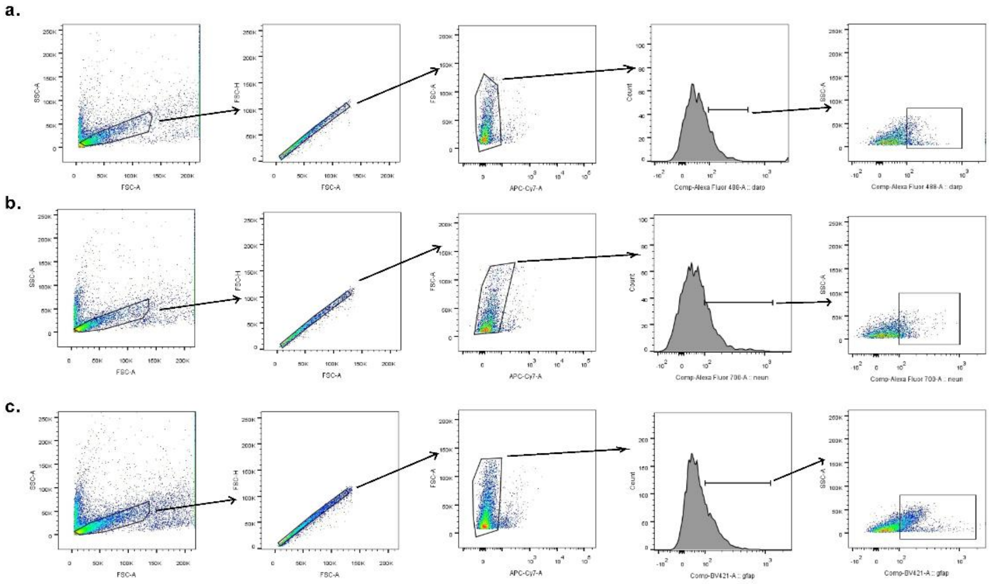

Supplementary figure 24. Gating strategies used for cell sorting. **(a)** Gating strategy to sort SPNs (Darpp32+). **(b)** Gating strategy to sort neurons (NeuN+). **(c)** Gating strategy to sort astrocytes (GFAP+).

Supplementary Table 1. Promoter sequences of *Mgll* gene (-1~-1000bp).

Monoglyceride lipase (Mus musculus; NCBI gene ID:23945)	GGGCCGCCCCCGCCGCCGAGCGGCTGGGGATAAAGTGGCGGCGCAGA<br>CGCCGCGCCCTATCGCTGCGCAGCCGGCCGCGCGCAGTAGTCTGGCTC<br>TAGCCCGCCGCAGACCCGGACCCGCGTGGTGCTCGGGGAACGTGACA<br>GCGGCGCTCGTGGCCCGGGTAACTGCCTCCCGCCCCTGCCTCAGGATA<br>GGAGCCGCCGCCAGGGGTCCCCTGCGCGCGTGTGCGTGCGCACGAGT<br>GTGCGCGCGTGTCAGCGTGTGCGCGTGAACGCGCCCCTCGCGCCCTCG<br>CACCAACCCCCAAACAGACTTGTGCCCGTCAACTGCATCTTCCCAGAC<br>CGGTGTGGGAACAACGGCCAGGTCCACGTCCTGTGTGGCGTGCCGATG<br>ACAGCCGCGAGGTTTCCTTCCCTAAGCGGTCGCGGGCGGGCAAGAGGC<br>AAAGTTTGTCGGAGAATCGGGGTGACTTTGCTCGGGGACCCGCATCTGC<br>GCGAATGCGCGGTGCCGCGGAGCGCGTCTCGCAGCAGCTCCGGGCTG<br>GGGACCGGCTGTCCCTCGCTCCCGGAACCCCTTCAGGGGTGTGTTCTGG<br>AAAAGTGGCGACATGAGCTAGAGCTGGAGCAGCAGGAGGGAGCAACT<br>CGCACCCCTGCAGCCCAGGCGGGGAGGGCGCGGACCCCGTGGTGCTG<br>CCCTGCGGCCGCGATGAGGGAACAGCCCTCGTTTGCCTGGCGCTGATA<br>CTCGAGGCTGTGGCGGTAGTGGAATGCAAAAGCCAAGACTAATGGAAA<br>CAGGTTTGTCATGGCATCTTTGGCATTCAGAAGGGAAAAATCGCTTGGG<br>GCGCGTCACCTACTTATTATGCCCCCTGAAACCCGCACAGTGGAGATTT<br>TAGGAGAGATGGGTCCTTGCAGCTGGGGCTTTGTACCCACGGAACTGA<br>CTGTAATTTCCGCTTGATTACAGCTAAAATAGTCTTTAAGATGAAGTTTC<br>CTGAGATGATTTAAAATAAACACAGATGGGAGGGGGGCTATACTGAGG<br>CACTTTAAAAAAATAAAGTGAGCATTTTCTTAAGGACAAACAGGACTTT<br>TCAATCTTGAAGGGGTTTGCAAGAGATGTATGTAATTTTAAAATATATAT<br>ATCAAATTGGGGTTTGGTGGGGTTTTTTTTGTTTGTTTGTTTTGTTTTTTGTTT<br>TTTGTTTTTTGTTTAATTGCCTAGAAAAGAAGCCACAGCCACAGTCCTGA<br>CTCAAAAAAGGCTTTCTTTCCTCCCATATGGACATAAGTGTATTTCTGAC<br>TTTAGAATATGTCCACCTTTCTTCCAAGGGCCTAAAGACCCCTGCAGG

Supplementary Table 2. Primers for qPCR.

**Mouse mRNA primers**	**Primer sequence (5' to 3')**
Actb(β-actin)-F	TTCGCGGGCGACGAT
Actb(β-actin)-R	CATCTTTTCACGGTTGGCCT
Cnr1(CB1 receptor)-F	GCTGCAATCTGTTTGCTCAG
Cnr1(CB1 receptor)-R	TTGCCATCTTCTGAGGTGTG
Faah(FAAH)-F	GCATGAGATTGAGATGTATCGCC
Faah(FAAH)-R	GTGTTCCATCTGGGCATCGT
Napepld(NAPE-PLD)-F	CCCGAATGTGCTCAGATGGC
Napepld(NAPE-PLD)-R	CCATTTCCACCATCAGCGTC
Mgll(MAGL)-F	CTGGGGATAAAGTGGCGGC
Mgll(MAGL)-R	CAAAGATGAGGGCCTTGGGT
Dagla(DAGLα)-F	TCGACCTCAAGAACTCGCAC
Dagla(DAGLα)-R	CAGGAAGTGACGGCGGATAG
Daglb(DAGLβ)-F	AAGCGGCCAGATACATTCAC
Daglb(DAGLβ)-R	GGATAAGCGACACGACAAAG
Abhd6-F	TGGATTCTCCGCACACAAGG
Abhd6-R	AACTTGCCCCACTATGGACAG
Abhd12-F	CGCTGGGCAGACGAAAGA
Abhd12-R	GGGCATTCTTCCACCAGACA

Supplementary Table 3. Primers for ChIP-qPCR.

**Mouse mRNA primers**	**Primer sequence (5' to 3')**
ChIP1-F	TGTCGGAGAATCGGGGTGAC
ChIP1-R	CCTGAAGGGGTTCCGGGAG
ChIP2-F	GGAAAAGTGGCGACATGAGC
ChIP2-R	CCACAGCCTCGAGTATCAGC
