# Supplementary material for: Endocannabinoid signaling regulates the reinforcing and psychostimulant effects of ketamine in mice
Source: Nat Commun. 2020 Nov 24;11:5962. doi: 10.1038/s41467-020-19780-z (PMC7686380; doi:10.1038/s41467-020-19780-z)
Supplement: Supplementary file 3 — Description of Additional Supplementary Files [file 41467_2020_19780_MOESM3_ESM.pdf]

Description of Additional Supplementary Files

Supplementary Data 1. Differential lipids between ketamine and saline groups.

Supplementary Data 2. Original data of lipidome detection in five brain regions. ACe, central amygdaloid nucleus.

CPu, caudate nucleus and putamen. NAc, nucleus accumbens. PFC, prefrontal cortex. Hipp, hippocampus. FA, fatty acid; MG, monoacylglycerol; DG, diacylglycerol; TG, triacylglycerol; CL, cardiolipin; LPA, lysophosphatidic acid; LPC, lysophosphatidylcholine; LPE, lysophosphatidylethanolamine; PA, phosphatidic acid; PAe, ether phosphatidic acid; PC, phosphatidylcholine; PCe, ether phosphatidylcholine; PE, phosphatidylethanolamine; PEe, ether phosphatidylethanolamine; PG, phosphatidylglycerol; GM3, monosialodihexosylganglioside; PGP, ; PI, phosphatidylinositol; PIe, ether phosphatidylinositol; PIP2, Phosphatidylinositol bisphosphate; PS, phosphatidylserine; PSe, ether phosphatidylserine; Cer, ceramide; CerP, ceramide 1-phosphate; HexCer, hexosylceramide; LacCer, lactosylceramide; SM, sphingomyelin; dhSM, dehydrosphingomylin; Sulf, sulfatides; Sulf(2OH), 2-hydroxy N-acyl sulfatide; PGe, ether phosphatidylglycerol; PIP, Phosphatidylinositol phosphate.

Supplementary Data 3. List of the primary and secondary antibodies.
